# Fascia lata allograft versus subepithelial connective tissue graft for peri-implant mucosal augmentation at implant placement: a randomized pilot clinical trial

**DOI:** 10.1186/s12903-026-08829-y

**Published:** 2026-06-23

**Authors:** Mohamed H. Helal, Hoda M. Elguindy, Sahar F. Ghoraba, Malak Y. Shoukheba

**Affiliations:** Department of Oral Medicine, Periodontology, Oral Diagnosis and Radiology, Faculty of Dentistry, Tanta University, Tanta, Egypt

## Abstract

**Objectives:**

To compare fascia lata allograft (FLA) and subepithelial connective tissue graft (SCTG) for peri-implant mucosal augmentation performed simultaneously with implant placement.

**Materials and methods:**

This prospective, randomized, examiner-blinded pilot clinical trial enrolled 16 patients requiring single-tooth implant placement with a thin peri-implant soft-tissue phenotype. Participants were randomly allocated (1:1) to SCTG or FLA. The primary outcome was peri-implant mucosal thickness (PMT) measured by using a customized stent-guided transmucosal method at standardized buccal reference points. Secondary outcomes included keratinized mucosal width (KMW), postoperative pain (VAS), and modified wound healing index (MWHI). Follow-up was 24 weeks.

**Results:**

Both groups demonstrated significant improvements in PMT and KMW over time. Between-group differences were generally non-significant, except localized PMT differences at the intermediate reference level, favoring SCTG at later visits. FLA showed lower postoperative pain at selected time points. Healing outcomes were comparable.

**Conclusion:**

Both SCTG and FLA were effective for peri-implant mucosal augmentation. FLA may represent a viable alternative that avoids donor-site morbidity. Larger long-term trials are required.

**Clinical relevance:**

FLA can be considered a viable alternative for enhancing peri-implant mucosal thickness around simultaneously placed dental implants.

**Trial registration:**

ClinicalTrials.gov NCT04679922, registered on December 22, 2020, and retrospectively registered.

**Graphical Abstract:**

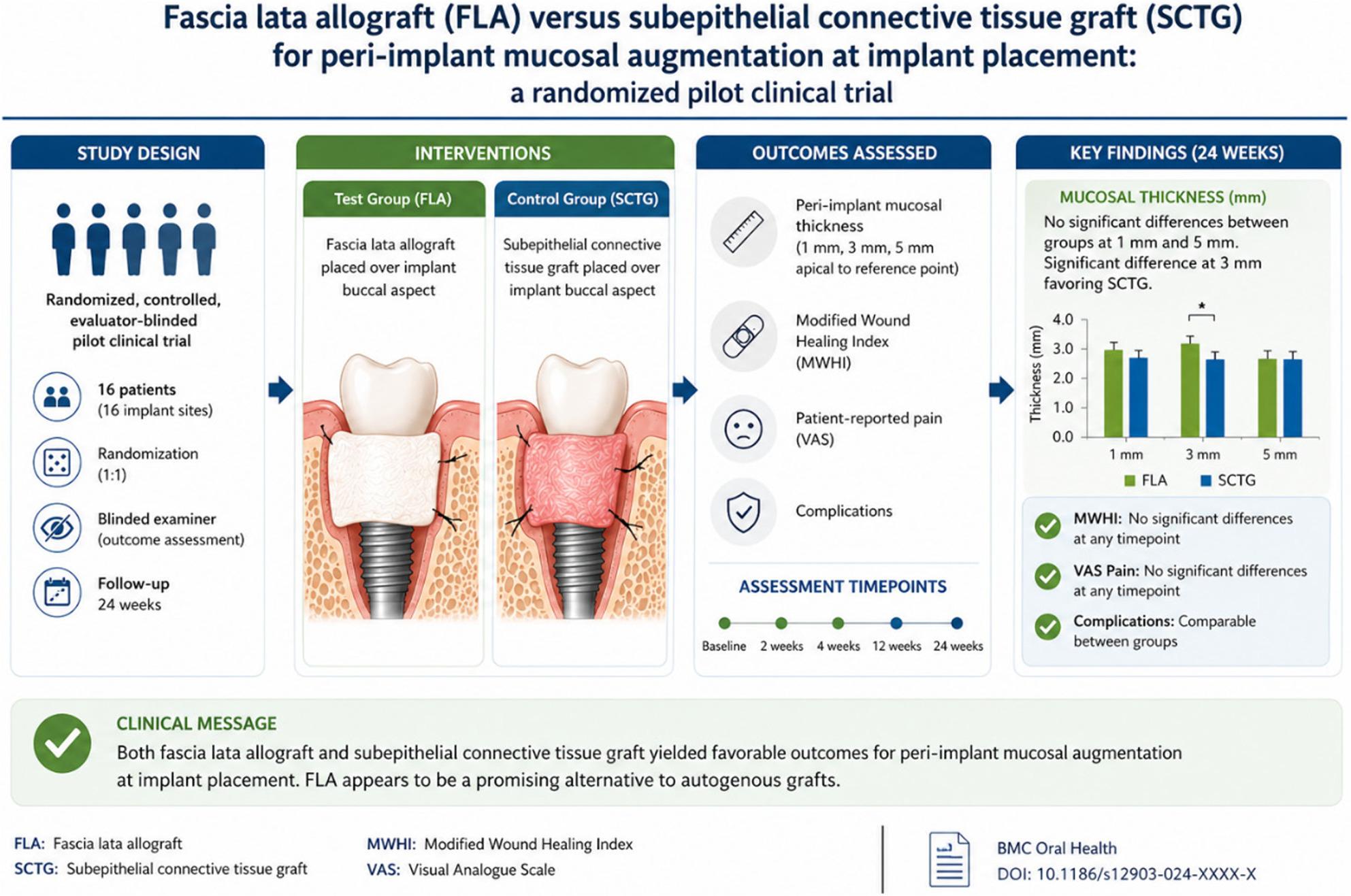

## Introduction

Peri-implant mucosal thickness (PMT) has been increasingly recognized as an important factor influencing peri-implant tissue health, marginal bone stability, and esthetic outcomes around dental implants [1, 2]. Previous prospective clinical studies have demonstrated that thin peri-implant mucosa may be associated with greater crestal bone remodeling. In contrast, increased soft-tissue thickness may contribute to improved peri-implant tissue stability [1–3]. In addition, soft-tissue augmentation performed simultaneously with immediate implant placement may help preserve peri-implant contours and improve long-term esthetic integration [4].

Autogenous subepithelial connective tissue grafts (SCTGs) are widely considered the gold standard for peri-implant soft-tissue augmentation due to their predictable clinical outcomes and favorable volumetric stability [5, 6]. However, SCTG harvesting requires a second surgical donor site, usually the palate, which may increase operative time and postoperative morbidity. Previous studies have reported complications such as pain, bleeding, delayed healing, and patient discomfort at the donor area [7, 8]. Furthermore, the quantity and quality of donor tissue may be limited in certain patients or when multiple sites require augmentation [7].

To overcome these limitations, several soft-tissue substitutes have been introduced, including acellular dermal matrix (ADM), collagen matrices, amniotic membrane, fibrin-based scaffolds, and synthetic polymeric biomaterials [9]. These substitutes aim to reduce donor-site morbidity while maintaining acceptable clinical outcomes. However, currently available materials differ in their biological behavior, structural integrity, volumetric stability, and long-term clinical performance.

Human fascia Lata allograft (FLA) is an allogenic acellular collagen-rich matrix that has been proposed as a soft-tissue graft substitute in oral regenerative procedures. During processing, cellular and antigenic components are removed while preserving the extracellular collagen scaffold, which may support host fibroblast migration, vascular ingrowth, and soft-tissue integration after placement [10]. Potential clinical advantages of FLA include broad availability, reduced patient morbidity, standardized processing, and elimination of palatal tissue harvesting.

Previous reports have described the use of fascia lata grafts in oral and implant-related applications, including bone regeneration adjacent to implants, ridge augmentation, and soft-tissue reconstructive procedures [10–13]. Moreover, in vitro investigations have demonstrated that fascia lata matrices may function as biologically compatible scaffolds for fibroblast attachment and proliferation [13]. Nevertheless, high-quality clinical evidence directly comparing FLA with SCTG for peri-implant mucosal augmentation remains scarce.

Therefore, the present randomized pilot clinical trial aimed to compare the clinical effectiveness of fascia lata allograft and autogenous subepithelial connective tissue graft for peri-implant mucosal augmentation performed simultaneously with implant placement. Despite the increasing interest in biomaterial substitutes for peri-implant soft-tissue augmentation, clinical evidence regarding the use of fascia lata allograft remains limited, particularly in randomized controlled clinical settings directly comparing FLA with autogenous connective tissue grafts. Therefore, the present pilot randomized clinical trial was designed to test the hypothesis that FLA may provide peri-implant soft-tissue augmentation outcomes comparable to SCTG while reducing postoperative morbidity associated with donor-site harvesting. The primary outcome was peri-implant mucosal thickness, while secondary outcomes included keratinized mucosal width, wound healing, postoperative pain, and short-term clinical complications.

## Materials and methods

### Study design and ethical approval

This study was designed, conducted, and reported in accordance with the CONSORT 2010 statement for randomized clinical trials. The investigation was a prospective, randomized, examiner-blinded, active-controlled pilot clinical trial with a 1:1 allocation ratio. The study protocol was approved by the Research Ethics Committee, Faculty of Dentistry, Tanta University, Egypt (Approval No. OMPDR-07-19-5). All procedures were performed in accordance with the Declaration of Helsinki for research involving human participants. The trial was registered at ClinicalTrials.gov (NCT04679922).

### Sample size and pilot trial rationale

The sample size was estimated according to the clinical trial conducted by Hutton et al. [14]. Based on their reported data, eight participants per group were considered sufficient to generate preliminary comparative estimates of treatment effect and feasibility outcomes. Because the present study was designed as a pilot randomized clinical trial, the sample size was considered appropriate for hypothesis generation and planning future definitive studies.

### Study population

Sixteen systemically healthy patients (10 females and 6 males), aged 30–55 years, requiring single-tooth implant placement in the maxillary anterior or premolar region, were recruited from the outpatient clinic of the Department of Periodontology, Faculty of Dentistry, Tanta University.

### Inclusion criteria

Participants were eligible if they fulfilled the following criteria:

- Good general health with no contraindication to periodontal surgery.
- Single missing tooth in the maxillary anterior or premolar region.
- Thin mucosal phenotype (< 2 mm bucco-palatal thickness).
- Adequate oral hygiene and compliance with maintenance visits.
- Sufficient bone volume for implant placement with simultaneous soft-tissue augmentation.

### Exclusion criteria

Patients were excluded if they had any of the following:

- Smoking > 10 cigarettes/day.
- Severe hematologic disorders.
- Uncontrolled infections or systemic disease impairing healing.
- Active periodontitis.
- Previous head and neck radiotherapy.
- Current or previous amino-bisphosphonate therapy.
- Pregnancy or lactation.
- Acute infection at the implant site.
- Poor oral hygiene or inability to attend follow-up visits.

All participants were informed about the study objectives and signed written informed consent before enrollment.

### Randomization and allocation concealment

Participants were randomly allocated into two treatment groups using a computer-generated random sequence prepared by an independent investigator not involved in treatment or outcome assessment. Allocation concealment was performed using sequentially numbered sealed opaque envelopes opened immediately before surgery.

The examiner responsible for postoperative measurements remained blind to treatment allocation throughout the study period.

### Surgical guide fabrication

Cone-beam computed tomography (CBCT) was obtained for all patients to determine ridge dimensions and available bone volume before implant placement [15]. Diagnostic casts were digitized using an extraoral optical scanner, and STL files were obtained according to Almog et al. [16].

**Fig. 1:**
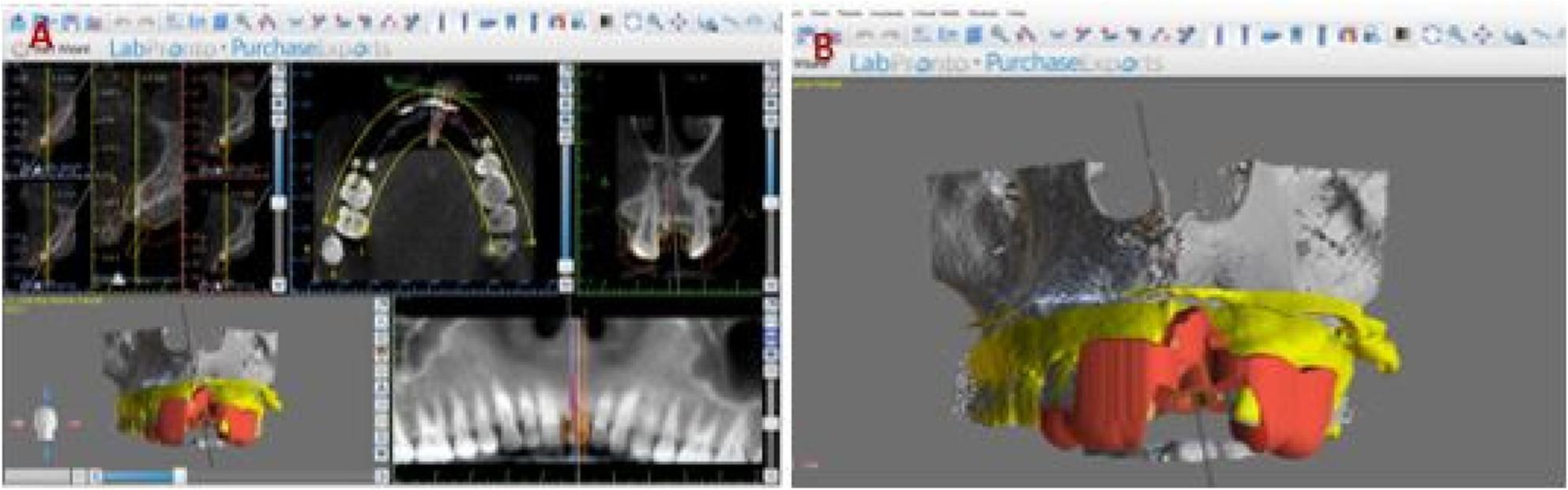
**A** Screenshot from Blue Sky Plan software demonstrating importation of DICOM files for virtual implant planning. Osteotomy positioning was planned based on the proposed implant site, considering the mesiodistal space, occlusal plane, and inter-occlusal clearance. **B** STL file of the diagnostic cast superimposed on the CBCT-derived bone model for guided surgical planning.

The DICOM files obtained from CBCT were merged with STL files using treatment-planning software (Blue Sky Plan version 2.19, Blue Sky Bio, LLC, Grayslake, IL, USA). Customized surgical guides were fabricated using a 3D printer (Any Cubic Photon 3D Printer, Shenzhen, Guangdong, China) (Fig. 1). Each guide included three standardized channels at the mid-buccal aspect of the edentulous ridge to allow reproducible measurement of peri-implant mucosal thickness at predetermined vertical reference points.

### Interventions

#### Group I (control group): SCTG

**Fig. 2:**
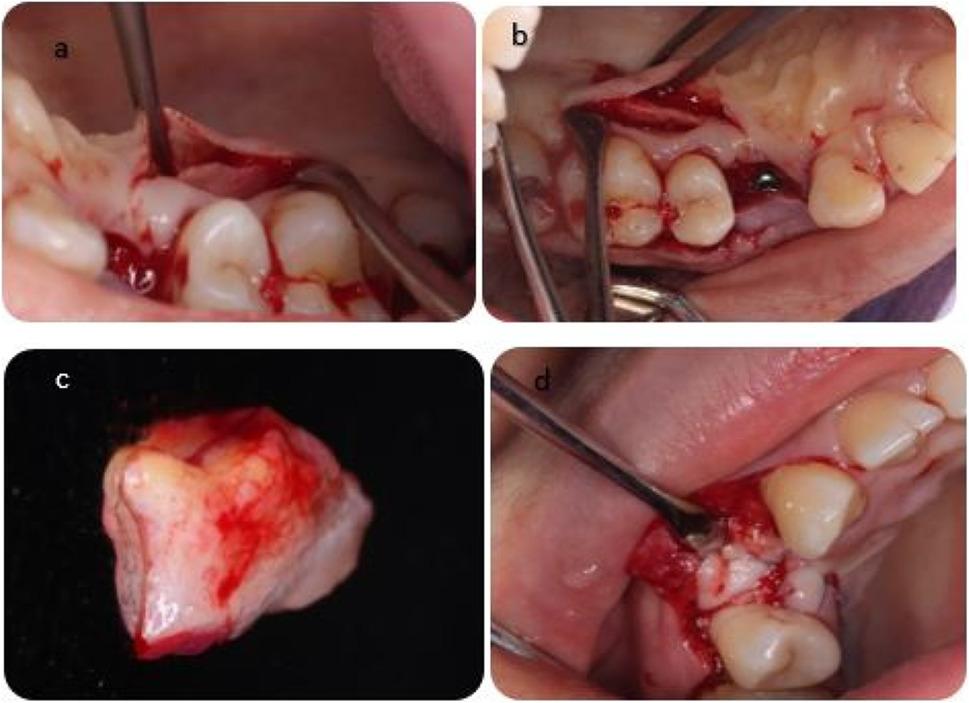
**A**, **B** Harvesting of autogenous subepithelial connective tissue graft (SCTG) from the palatal donor site using a single-incision technique extending from the first premolar to the distal aspect of the first molar. **C**, **D** Harvested graft adapted and stabilized over the recipient implant site using cross sutures with non-absorbable 5-0 sutures anchored to the buccal periosteum and palatal flap.

Patients received implant placement with simultaneous augmentation using an autogenous subepithelial connective tissue graft harvested from the palate according to the technique described by Lorenzana and Allen [17] (Fig. 2).

#### Group II (Test Group): FLA

**Fig. 3:**
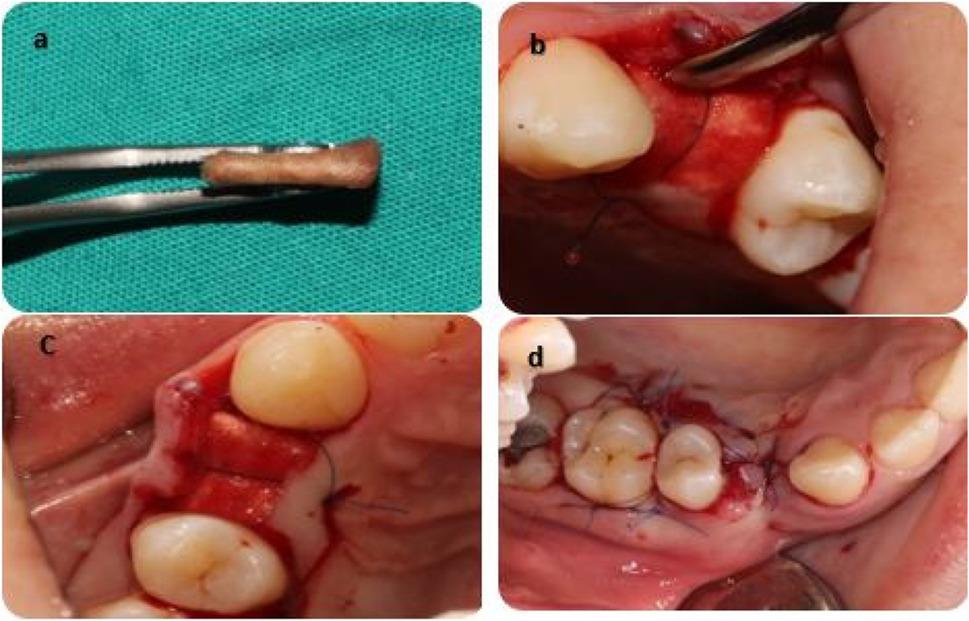
**A**–**D** Fascia lata allograft (FLA) trimmed to the appropriate dimensions and adapted over the coronal and buccal aspects of the alveolar ridge. The graft was stabilized using non-absorbable 5-0 sutures anchored to the buccal periosteum and palatal flap

Patients received implant placement with simultaneous augmentation using fascia lata allograft (FLA Maxxeus Dental, Ohio, USA), trimmed according to recipient site dimensions and hydrated according to the manufacturer’s instructions (Fig. 3).

### Surgical procedure

The same experienced operator performed all surgical procedures under strict aseptic conditions and local anesthesia using articaine 4% with 1:100,000 epinephrine (ARTINIBSA, Inibsa Dental S.L.U., Barcelona, Spain).

Preoperative antibiotic prophylaxis consisted of amoxicillin 2 g orally 1 h before surgery, or clindamycin 600 mg in penicillin-allergic patients, according to commonly accepted oral implant prophylaxis regimens.

**Fig. 4:**
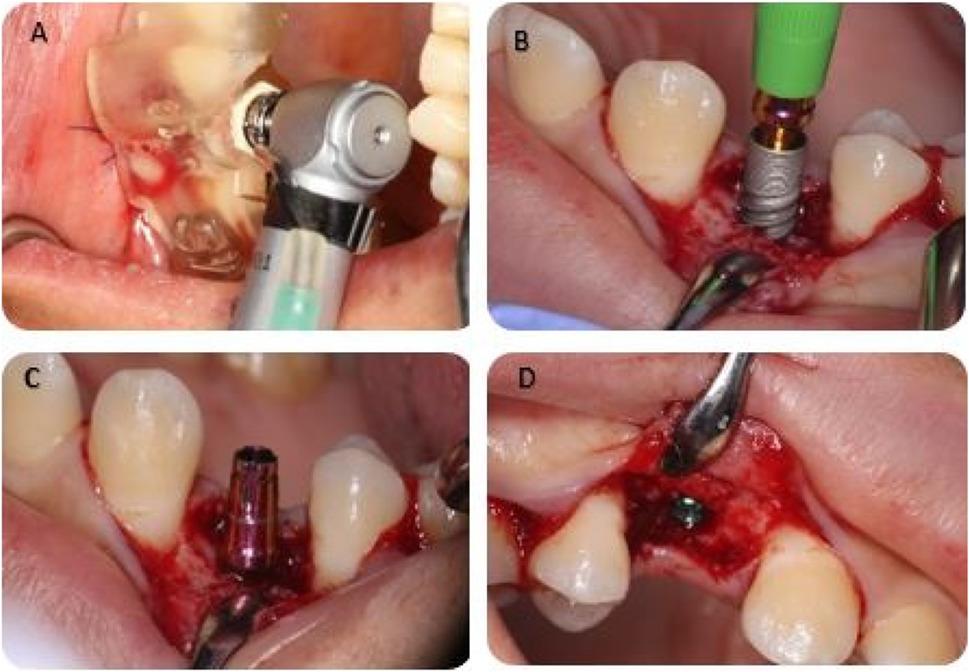
**A** Initial implant osteotomy preparation using a pilot drill. **B**, **C** Dental implant placement in parallel alignment with adjacent teeth. **D** Implant secured with cover screw following insertion

A paracrestal incision with vertical releasing incisions when required was performed. Implant osteotomy and placement were completed according to the manufacturer’s instructions (ROOTT Implants, Switzerland). A combined full-thickness/partial-thickness flap design was used to prepare the recipient bed (Fig. 4).

The graft material was adapted over the coronal and buccal ridge area and stabilized using 5 − 0 polyamide sutures (Seralon^®^, Resorba, Germany), selected for graft fixation. Flap closure was completed using 5 − 0 polypropylene sutures (Prolene^®^), selected for soft-tissue adaptation and reduced tissue drag (Fig. 3).

### Postoperative care

Ibuprofen 600 mg was prescribed as needed for pain control for up to 7 days unless contraindicated. Patients were instructed to rinse twice daily with 0.12% chlorhexidine for 2 weeks and to avoid brushing the surgical site during the early healing phase. Sutures were removed after 14 days. Definitive prosthetic restoration was delivered after 6 months.

### Outcome measures

#### Primary outcome: Peri-Implant Mucosal Thickness (PMT)

PMT was measured at the mid-buccal aspect using a customized stent-guided transmucosal probing technique at three standardized vertical reference points (1, 3, and 5 mm apical to the planned mucosal margin).

**Fig. 5:**
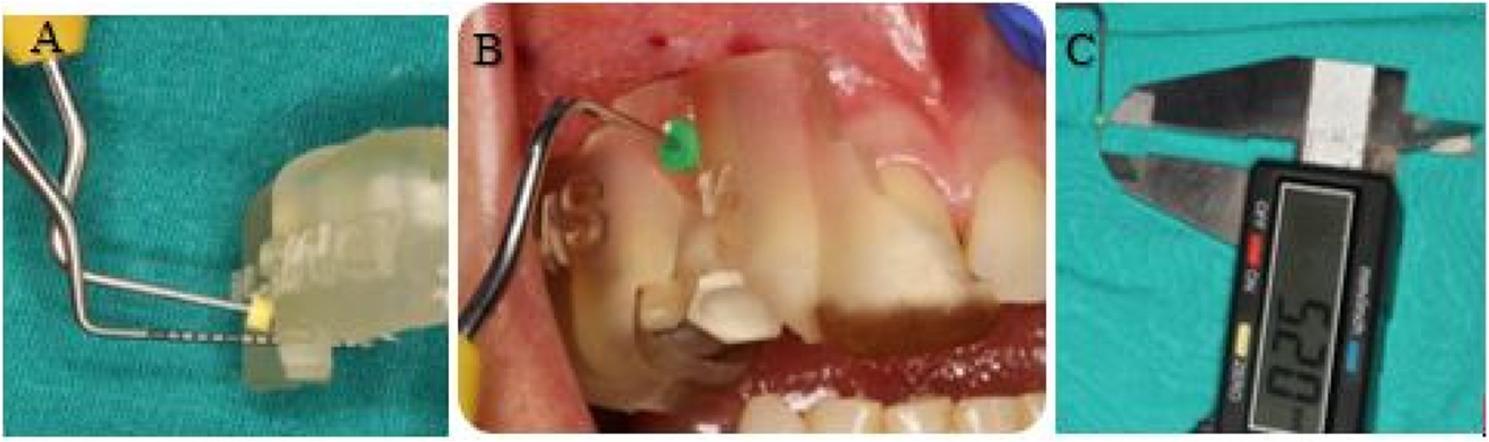
**A**, **B** Assessment of peri-implant mucosal thickness using the customized surgical guide. **A** shows the measurement of the soft-tissue thickness through the guide channel using a UNC-15 periodontal probe/endodontic spreader. **B** shows measurement of the underlying bone level through the same guide channel. **C** The difference between soft-tissue and bone measurements was calculated using a digital caliper and recorded in millimeters (mm)

A UNC-15 periodontal probe and endodontic spreader (DENTSPLY MAILLEFER, Switzerland) were inserted through the guide channels to record soft-tissue and bone contact levels (Fig. 5A, B). The difference between the two readings represented mucosal thickness. Measurements were recorded using a digital Vernier caliper (BESTOOL KANON, Tokyo, Fig. 5C). Measurements were obtained preoperatively, immediately after surgery (baseline), and at 4, 8, 12, and 24 weeks.

### Examiner calibration

A single, blind, calibrated examiner performed all clinical measurements. Before study initiation, repeated pilot measurements were conducted on non-study cases to standardize the measurement technique. Duplicate measurements within ± 0.5 mm were considered clinically acceptable. Formal intra-examiner reliability statistics, such as intraclass correlation coefficients (ICC) were not calculated, which should be considered a methodological limitation of the present pilot trial.

#### Secondary outcomes

1. *Keratinized Mucosal Width (KMW)*: measured at the mid-buccal aspect at baseline and follow-up visits.
2. *Postoperative Pain*: recorded using a 100-mm visual analogue scale (VAS) at 1 h, 6 h, 12 h, day 1, day 3, day 7, and day 15.
3. *Modified Wound Healing Index (MWHI)*: assessed postoperatively only according to Huang et al. [18] at appropriate healing visits.
4. *Complications*: infection, graft exposure, implant failure, or adverse healing events.

### Statistical analysis

Data was analyzed using SPSS software (IBM Corp., USA). Continuous variables were expressed as mean ± standard deviation when normally distributed and median (interquartile range) when non-normal. Between-group comparisons were performed using independent t-tests or Mann–Whitney U tests, as appropriate. Within-group repeated comparisons were analyzed using paired tests. Categorical variables were analyzed using chi-square or Fisher’s exact tests. Because this was an exploratory pilot trial, multiplicity adjustment for multiple comparisons was not prespecified. Therefore, statistically significant findings were interpreted cautiously as preliminary and hypothesis-generating. A two-sided p-value < 0.05 was considered statistically significant. Because of the exploratory pilot nature of the study and the limited sample size, the findings should be interpreted as preliminary and hypothesis-generating rather than definitive confirmatory evidence.

## Results

**Fig. 6:**
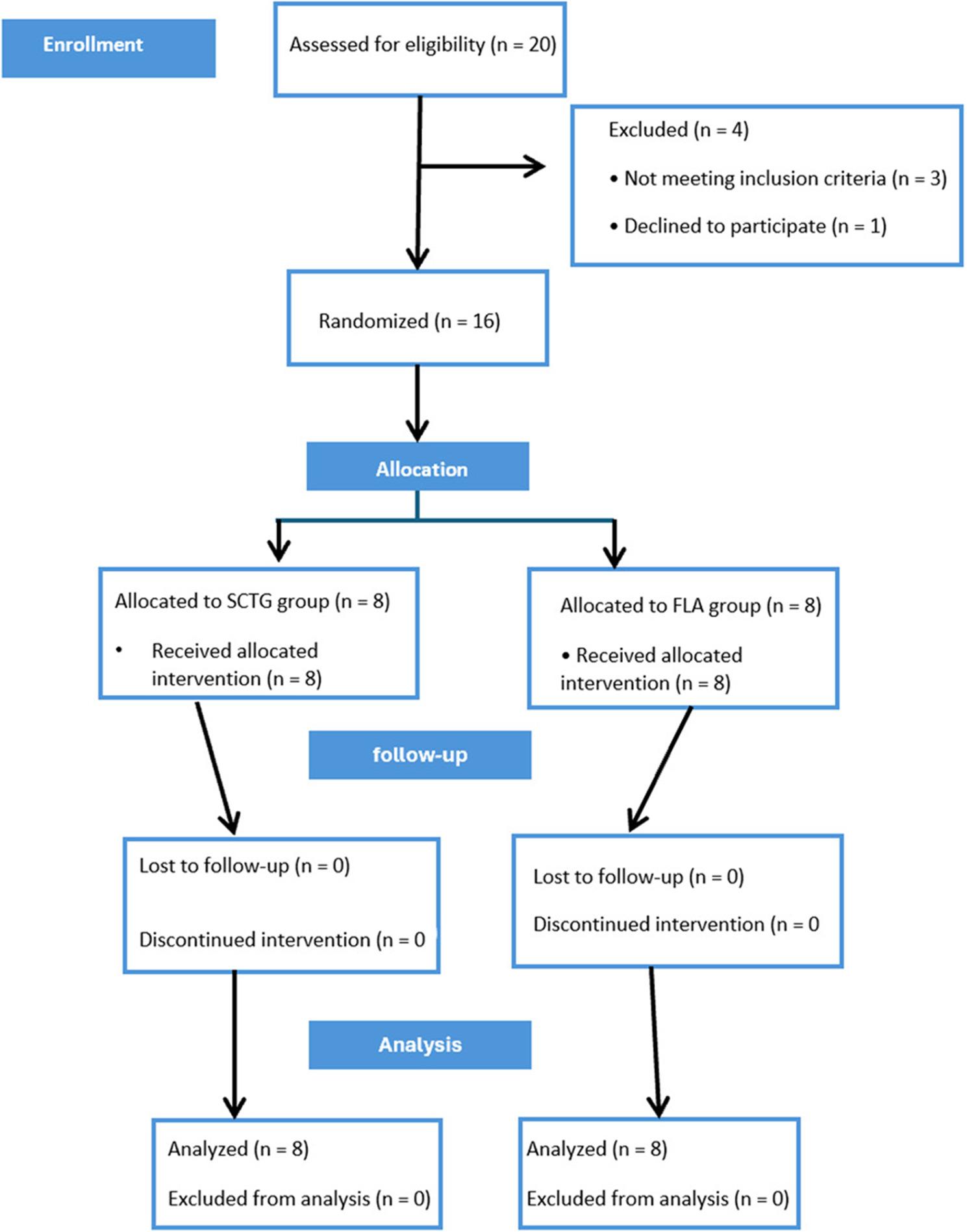
CONSORT flow diagram of participant recruitment, randomization, allocation, follow-up, and final analysis

The participant flow diagram is presented in Fig. 6. All sixteen enrolled participants completed the 24-week follow-up period and were included in the final analysis. No implant failures, graft rejection, infection, or other major postoperative complications were observed throughout the study period.

### Peri-implant Mucosal Thickness (PMT)

At baseline and preoperative measurements, no statistically significant differences were observed between the two groups at any of the evaluated reference levels (*p* > 0.05, Table 1), confirming comparability between groups before treatment.

**Table 1:** Comparison of peri-implant mucosal thickness (PMT) measurements at 1 mm, 3 mm, and 5 mm, apical to the reference point in the two treatment groups

Time Point	1 mm Apical SCTG Mean ± SD	FLA Mean ± SD	*p*-value	3 mm Apical SCTG Mean ± SD	FLA Mean ± SD	*p*-value	5 mm Apical SCTG Mean ± SD	FLA Mean ± SD	*p*-value
Baseline	4.47 ± 0.45	4.11 ± 0.19	0.059	4.84 ± 0.48	4.28 ± 0.70	0.086	4.78 ± 0.49	4.70 ± 0.80	0.817
4 weeks	4.41 ± 0.40	3.98 ± 0.22	0.017*	4.70 ± 0.48	4.17 ± 0.70	0.100	4.58 ± 0.44	4.36 ± 0.59	0.419
8 weeks	4.14 ± 0.48	3.86 ± 0.23	0.156	4.45 ± 0.50	4.01 ± 0.57	0.117	4.41 ± 0.43	4.27 ± 0.67	0.618
12 weeks	4.06 ± 0.38	3.71 ± 0.23	0.042*	4.26 ± 0.41	3.74 ± 0.24	0.007**	4.22 ± 0.48	4.12 ± 0.60	0.707
24 weeks	3.89 ± 0.31	3.55 ± 0.32	0.046*	3.98 ± 0.30	3.55 ± 0.22	0.006**	4.01 ± 0.41	3.90 ± 0.49	0.627
Gain	1.67 ± 0.58	1.51 ± 0.43	0.515	2.10 ± 0.32	1.68 ± 0.45	0.048*	1.59 ± 0.43	1.45 ± 0.57	0.584

Legend: *PMT* Peri-implant mucosal thickness, *SCTG* Subepithelial connective tissue graft, *FLA* Fascia lata allograft, *SD* Standard deviation

All *p*-values were calculated using the independent samples t-test

* Statistically significant at *p* < 0.05

** Highly statistically significant at *p* < 0.01

#### PMT at 1 mm apical to the reference point

At the 4-week follow-up, a statistically significant between-group difference was observed. However, this difference was not maintained consistently over time. At the final 24-week evaluation, the mean PMT gain was 1.67 ± 0.58 mm in the SCTG group and 1.51 ± 0.43 mm in the FLA group, with no statistically significant difference between groups (*p* = 0.515).

#### PMT at 3 mm apical to the reference point

No statistically significant differences were identified at 4 weeks (*p* = 0.100) or 8 weeks (*p* = 0.170). However, statistically significant differences were observed at 12 weeks (*p* = 0.007) and 24 weeks (*p* = 0.006), in favor of the SCTG group. At 24 weeks, the mean PMT gain was 2.10 ± 0.32 mm for the SCTG group and 1.68 ± 0.45 mm for the FLA group. This difference was statistically significant (*p* = 0.048).

#### PMT at 5 mm apical to the reference point

No statistically significant differences were found between groups at any follow-up time point. At the 24-week evaluation, the mean PMT gain was 1.59 ± 0.43 mm in the SCTG group and 1.45 ± 0.57 mm in the FLA group (*p* = 0.584).

### Keratinized Mucosal Width (KMW)

Both treatment groups demonstrated a statistically significant increase in keratinized mucosal width (KMW) from baseline to 24 weeks (*p* < 0.05). The mean KMW gain in the SCTG group was 1.13 ± 0.38 mm, whereas the FLA group demonstrated a mean gain of 1.06 ± 0.32 mm. However, the difference in KMW gain between groups was not statistically significant (*p* > 0.05, Table 2).

**Table 2:** Changes in keratinized mucosal width (KMW) from baseline to 24 weeks in the two treatment groups

Variable	SCTG Group (*n* = 8) Mean ± SD	FLA Group (*n* = 8) Mean ± SD	t-value	*p*-value
Baseline KMW (mm)	5.31 ± 1.25	5.38 ± 1.03	-0.109	0.915
24-week KMW (mm)	6.44 ± 1.29	6.44 ± 0.90	0.000	1.000
Gain in KMW (mm)	1.13 ± 0.38	1.06 ± 0.32	-0.357	0.727

Legend: *KMW* Keratinized mucosal width, *SCTG* Subepithelial connective tissue graft, *FLA* Fascia lata allograft, *SD* Standard deviation, *t* Independent samples t-test, *n* Number of sites

Both groups demonstrated a statistically significant within-group increase in KMW from baseline to 24 weeks (*p* < 0.001)

Between-group differences were not statistically significant (*p* > 0.05)

### Postoperative pain assessment

Regarding patient-reported pain outcomes, the FLA group (Group II) generally demonstrated lower visual analogue scale (VAS) scores than the SCTG group (Group I) during the early postoperative period. Statistically significant reductions in pain were observed in favor of the FLA group at the 6-hour, 12-hour, day 3, and day 7 assessments. No statistically significant differences were found between groups at 1 h, day 1, or day 15 (*p* > 0.05, Table 3).

**Table 3:** Postoperative pain scores (VAS) at different follow-up periods

Time Point	SCTG Group Mean Rank	FLA Group Mean Rank	Z value	*p*-value
1 h	9.19	7.81	-0.623	0.533
6 h	10.69	6.31	-1.925	0.054
12 h	11.94	5.06	-3.025	0.002*
Day 1	10.25	6.75	-1.555	0.120
Day 3	12.13	4.88	-3.218	0.001**
Day 7	11.88	5.13	-3.057	0.002*
Day 15	10.00	7.00	-1.861	0.063

Legend: *VAS* Visual analogue scale, *SCTG* Subepithelial connective tissue graft, *FLA* Fascia lata allograft, *Z* Z statistic of the Mann–Whitney U test for non-parametric data

* Statistically significant at *p* < 0.05

** Highly statistically significant at *p* < 0.01

### Postoperative wound healing

Healing outcomes were favorable in both treatment groups. The Modified Wound Healing Index (MWHI) scores indicated uneventful soft-tissue healing with no evidence of clinically relevant delayed healing or adverse tissue response. No statistically significant differences were detected between the SCTG group and the FLA group at any postoperative follow-up visit (*p* > 0.05, Table 4).

**Table 4:** Modified wound healing index (MWHI) scores during postoperative follow-up in the two treatment groups

Follow-up	SCTG Group (*n* = 8) Score 1 n (%)	Score 2 *n* (%)	Mean Score	FLA Group (*n* = 8) Score 1 n (%)	Score 2 *n* (%)	Mean Score	χ²	*p*-value
4 weeks	4 (50.0)	4 (50.0)	1.50	6 (75.0)	2 (25.0)	1.25	2.667	0.102
8 weeks	7 (87.5)	1 (12.5)	1.12	8 (100.0)	0 (0.0)	1.00	0.750	0.386
12 weeks	8 (100.0)	0 (0.0)	1.00	8 (100.0)	0 (0.0)	1.00	—	—
24 weeks	8 (100.0)	0 (0.0)	1.00	8 (100.0)	0 (0.0)	1.00	—	—

Legend: *MWHI* Modified wound healing index, *SCTG* Subepithelial connective tissue graft, *FLA* Fascia lata allograft, *χ²* Chi-square test, *n* Number of sites

No statistically significant differences were observed between groups at any follow-up visit (*p* > 0.05)

### Overall interpretation

Both SCTG and FLA produced clinically favorable increases in peri-implant mucosal thickness and keratinized mucosal width over the 24-week follow-up period. Between-group differences were generally limited, with SCTG demonstrating greater thickness gain only at the intermediate (3 mm) reference level during later follow-up. The FLA group showed reduced postoperative discomfort at selected early healing intervals.

## Discussion

Peri-implant mucosal phenotype is increasingly recognized as an important determinant of peri-implant tissue stability and esthetic success. Previous prospective clinical trials demonstrated that thicker peri-implant soft tissue may be associated with reduced crestal bone remodeling compared with thin mucosa [1, 2]. In addition, a mucosal thickness of at least 1 mm has been associated with reduced buccal mucosal recession, particularly in the esthetic zone [19]. Although the precise influence of keratinized mucosa on implant survival remains debated, several studies have suggested that adequate keratinized tissue may contribute to improved plaque control, peri-implant health, and patient comfort [20–22].

Accordingly, the primary objective of the present randomized pilot clinical trial was to evaluate peri-implant mucosal thickness (PMT) changes following simultaneous implant placement using either autogenous subepithelial connective tissue grafts (SCTGs) or fascia lata allograft (FLA). SCTG remains the reference standard because of its favorable clinical predictability and volumetric stability; however, it requires a second surgical donor site and may be associated with bleeding, pain, delayed healing, and patient morbidity [7, 8]. Furthermore, donor tissue quantity may be limited in some clinical situations [7].

FLA represents an allogenic acellular collagen-rich matrix proposed as a soft-tissue substitute. It has been described as biocompatible, flexible, and structurally stable, while eliminating the need for palatal harvesting [10, 23]. Processing methods aim to remove antigenic cellular components while preserving the extracellular collagen scaffold that may support fibroblast migration, vascularization, and tissue remodeling [10, 11, 14].

In the present study, both SCTG and FLA were clinically well tolerated, with no implant loss, graft rejection, or major postoperative complications during the 24-week follow-up period. Healing scores were comparable between groups, suggesting that both grafting materials were associated with favorable soft-tissue healing. These findings support the short-term clinical safety of FLA when used for peri-implant mucosal augmentation.

In this study, FLA was utilized as a grafting material substitute for host connective tissue for peri-implant soft tissue augmentation. This approach resulted in mild pain and edema. The surgical guide allowed us to place dental implants in an accurate position [24]. This surgical stent demonstrated a predictable outcome in measuring PMT, which is consistent with Hutton et al. [17], who measured PMT that was augmented with ADM or SCTGs over implants.

Importantly, our previous histological and histomorphometric investigation demonstrated favorable integration of FLA within peri-implant soft tissues, with newly formed fibrovascular tissue and significantly greater vimentin expression compared with SCTG after 3 months of healing, indicating active connective tissue remodeling and cellular repopulation (Helal et al., 2022) [25]. These observations may provide a biological explanation for the clinically acceptable soft-tissue performance observed in the present trial.

Regarding PMT outcomes, both groups demonstrated clinically meaningful increases in mucosal thickness over time. At the final 24-week evaluation, statistically significant differences were limited to the intermediate measurement level (3 mm), favoring SCTG. This isolated finding may reflect the known volumetric stability of autogenous connective tissue grafts, which contain native collagen bundles and viable connective tissue components. However, given the pilot nature of the present study, limited sample size, and multiple comparisons, this result should be interpreted cautiously and confirmed in larger, adequately powered trials.

Our findings may be partially comparable to those reported by Cairo et al. [26], who evaluated xenogeneic collagen matrix versus SCTG for peri-implant soft-tissue augmentation. However, direct comparison should be interpreted cautiously because the biomaterial investigated in their study differed substantially from the fascia lata allograft in biological origin, structural composition, remodeling behavior, and volumetric stability characteristics. Unlike xenogeneic collagen matrices, FLA represents a dense human-derived collagen scaffold with distinct biological integration properties and tissue remodeling kinetics. Similarly, previous clinical reports have described favorable soft-tissue augmentation outcomes using connective tissue grafts and collagen-based substitutes; however, the available evidence includes case reports and retrospective investigations in addition to randomized clinical trials [27–29]. Therefore, direct comparison across studies should be interpreted cautiously because of heterogeneity in study design and evidence level.

Both treatment groups also demonstrated significant increases in keratinized mucosal width (KMW) in 24 weeks, with no statistically significant difference between groups. These findings are in agreement with Abass et al. [30], who reported favorable peri-implant soft-tissue outcomes using acellular dermal matrix in comparison with SCTG. In contrast, Hutton et al. [17] reported greater dimensional loss with ADM, which may be related to manufacturing-associated shrinkage characteristics rather than a general limitation of all soft-tissue substitutes [31].

Regarding patient-reported outcomes, the FLA group exhibited significantly lower pain scores at selected early postoperative intervals. This is clinically relevant and likely attributable to the elimination of palatal harvesting. Similar reductions in postoperative discomfort with soft-tissue substitutes have been reported previously [32, 33]. Reduced morbidity may represent an important advantage of FLA in patients with low pain tolerance or when multiple augmentation procedures are required. The present study has several limitations that should be acknowledged. First, the sample size was modest because this investigation was designed as a pilot randomized clinical trial. Second, follow-up was limited to 24 weeks and does not permit definitive conclusionsregarding long-term volumetric stability. Third, this was a single-center study, which may limit generalizability. Finally, although previous histological evidence is available [25], histological evaluation was not performed within the current clinical trial.

Within these limitations, both SCTG and FLA were effective in increasing PMT and KMW during short-term follow-up. While SCTG demonstrated greater thickness gain at one intermediate level, FLA provided meaningful clinical benefits by reducing postoperative pain and eliminating donor-site morbidity. In addition, postoperative analgesic consumption was not quantitatively recorded during follow-up, which may have influenced patient-reported VAS pain outcomes. Therefore, FLA may represent a promising alternative for peri-implant soft-tissue augmentation in appropriately selected cases.

The present study has several limitations that should be acknowledged. First, the sample size was modest because this investigation was designed as a pilot randomized clinical trial. Second, follow-up was limited to 24 weeks and therefore does not permit definitive conclusions regarding long-term volumetric stability. Third, this was a single-center study, which may limit external generalizability. Fourth, postoperative analgesic consumption was not quantitatively recorded and may have influenced patient-reported pain outcomes. In addition, formal intra-examiner reliability statistics such as intraclass correlation coefficients were not calculated. Finally, although previous histological evidence is available [25], histological evaluation was not performed within the current clinical trial.

## Conclusions

Within the limitations of this randomized pilot clinical trial, both fascia lata allograft (FLA) and subepithelial connective tissue grafts (SCTGs) were effective in increasing peri-implant mucosal thickness and keratinized mucosal width when performed simultaneously with implant placement. Although SCTG demonstrated greater thickness gain at one intermediate measurement level, FLA showed favorable healing outcomes and reduced postoperative discomfort at selected time points.

Therefore, FLA may be considered a feasible and clinically promising alternative for peri-implant soft-tissue augmentation, particularly when avoidance of donor-site morbidity is desirable. Larger multicenter trials with longer follow-up periods are warranted to confirm these findings.

## Data Availability

The datasets generated and analyzed during the current study are not publicly available because of patient privacy and ethical restrictions, but are available from the corresponding author upon reasonable request.

## Abbreviations

SCTG: Subepithelial connective tissue graft
SCTGs: Subepithelial connective tissue grafts
FLA: Fascia lata allograft
PMT: Peri-implant mucosal thickness
KMW: Keratinized mucosal width
VAS: Visual analogue scale
MWHI: Modified wound healing index
ADM: Acellular dermal matrix
PLGA: Polylactic-co-glycolic acid
CBCT: Cone-beam computed tomography
SRP: Scaling and root planing
